# How personal values and critical dispositions support digital citizenship development in higher education students

**DOI:** 10.3389/fpsyg.2022.990518

**Published:** 2022-10-04

**Authors:** Gabriela Monica Assante, Nicoleta Laura Popa, Mariana Momanu

**Affiliations:** Educational Sciences Department, Alexandru Ioan Cuza University of Iași, Iași, Romania

**Keywords:** transversal competences, students, digital citizenship, personal values, critical thinking

## Abstract

The virtual environment’s expansion and role in young people’s lives accentuate the need for developing transversal competences such as digital citizenship. The process may be supported by personal resources like personal values and critical thinking dispositions. With this study on 536 young students’ students aged 18 to 26 (*M* = 20.85, *SD* = 1.60), we analysed the relationship between two adjacent personal values, universalism and self-direction, and students’ digital citizenship. Moreover, we examined the role of critical thinking dispositions, namely learning orientation, and cognitive integrity in supporting digital citizenship development. Following structural equation modelling (SEM) analyses, the results show that universalism and learning orientation significantly positively influence digital citizenship, whereas cognitive integrity has a negative effect. Further, personal values positively associate with critical thinking dispositions.

## Introduction

For more than two decades, the development of digital competences has been approached as transversal or soft competences to be embedded in the higher education curriculum as a specific response to the pressure of employability and economic growth in contemporary and future societies. Although higher education around the world has experienced major changes in the curriculum in this respect and remarkable progress has been made towards advancing the employability-oriented profiles of the graduates, transversal competences are still hindered in favour of a theoretical, content-based university curriculum (Oria, 2012). Alongside digital competences, transversal competences also address entrepreneurship, teamwork, creativity, communicativeness, critical thinking and the ability to cope with complexity and incertitude (Larsen, 2013; Sá and Serpa, 2018; Graczyk-Kucharska et al., 2020). An important note should be made around differences between skills and competences, with implications for defining and measuring each construct: whereas skills are acquired abilities, competences express the mobilisation of abilities and other additional resources (especially knowledge and critical understanding, values and attitudes) in specific professional or life contexts (Van der Velden, 2013). Thus, skills are to be considered and measured as elements enclosed within competences.

Recent EU higher education initiatives emphasise the critical role of universities in shaping more green and more digital economies [Council of the European Union, 2022a; Council recommendation on building bridges for effective European higher education cooperation, (Council of the European Union, 2022b)], and digital competences play a central part in this process. Thus, beyond ‘hard competences’ targeted as central learning outcomes of university study programmes, digital competences and other key transversal acquisitions have become a pivotal interest for teachers, learners, and researchers in higher education. Intensively studied in the last decades, digital competences among university students grow into relevant long-term assets. They are positively connected to work-related competences through self-esteem and self-regulated learning (Khampirat, 2021), to professional self-efficacy (Chonsalasin and Khampirat, 2022) and as to professional social capital (Chaker, 2020).

In a larger and diverse network of concepts (e.g., global competence and citizenship, digital competence for citizens, media literacy etc.), digital competences along with digital literacy underpin ‘digital citizenship’, a term that has entered the policy and academic discourse to stand for competent, confident and responsible or ethical use of technology (Ribble et al., 2004) based on respect for others and democratic values. Several empirical studies on digital citizenship in higher education have been conducted in recent years (e.g., Al-Zahrani, 2015; Kara, 2018; Takavarasha et al., 2018); however, coherent digital citizenship education in the university curriculum is most probably seen as a ‘natural’ outcome of efforts invested in developing students’ digital competences and, thus, is rather neglected in policy papers and programme contents. Given the profound changes undertaken by universities for better preparing students for future societies, it is reasonable to assume that digital citizenship will further enrich the meaning of digital competence development and fully enter the academic debate on transversal competences in higher education. While education programmes in this area are focused more on increasing students’ digital skills, public concern regarding the potential risks to youth online has prompted a quick response to provide internet safety education. The concept of digital citizenship comprises four different dimensions: media and information literacy, critical resistance, participation and engagement, and digital ethics (Choi et al., 2017). In this study, the critical perspective dimension of digital citizenship is explored, defined as the ability to approach different perspectives or to use the perspective of others in the development of new ideas (Sayer, 2009). This approach enables the decolonisation of knowledge by promoting thinking that challenges the status quo (Smith, 1999). In this context, users with advanced Critical Perspective see online activity as valuable for continuously comparing to traditional forms of engagement with new possibilities. Users can then transform the Internet from a neutral information communication/distribution technology tool into a tool that is potentially susceptible to biases as with all other human tools (Feenberg, 1991). For the Internet to provide greater understanding, it should not be a tool of authority but rather one that allows for exploring, exchanging, comparing and augmenting ideas. The digital space represents an area of individual development and the expression of values. Hence, Gazi (2016) defines digital citizenship as “a socially constructed set of practices and the norms of behaviours that facilitate individual development and protect social values in a digital society” (p. 139). Digital citizenship spans different areas of education and is not separated from the rest of the curriculum. Therefore, in supporting the development of digital citizenship, teaching and learning strategies must be established in close relationship with values and the development of higher-order thinking such as critical thinking (Al-Abdullatif and Gameil, 2020). In addition, to develop sustainable digital citizenship, values must be clearly defined for both the digital and physical environment (Ohler, 2011). Embracing values in virtual communities is useful to create a positive culture that will promote sustainable digital citizenship (Ghosn-Chelala, 2019). Citizenship in this sense is not only about acts of expression by young people in a digital sphere, which reflect their ethics (Bennett, 2008; Bennett and Segerberg, 2012), but also manifests in other ways. Youth may be keen to share their values and impact their peers’ attitudes through the digital environment. Nevertheless, educators also have an essential role in developing skills to enhance the problem-solving ability of students and competences to create persuasive media and strategically distribute it to their friends and respective communities (Gleason and Von Gillern, 2018).

In this dynamic, Schwartz’s theory of universal substance and structure of basic values (Schwartz, 1992, 2012) was considered as it is widely used in modern value frameworks and recognised for its explanatory power in relation with various individual and group attitudes and behaviours (Arieli et al., 2020; Russo et al., 2022). It provides a solid theoretical foundation for establishing hypotheses based on a person’s value system. According to Schwartz, values are general goals by which individuals guide their lives. In the context of guiding principles, appropriate values influence long-term behaviour in various contexts, e.g., social, personal or professional. In this way, fundamental values can help predict behaviour in various contexts. Every person has a different value hierarchy, meaning one value may be important to one person but not another. The theory delineates 10 different values, with each determined by distinct motivation aims (Schwartz, 2005, 2012; Borg et al., 2015). Values are empirically associated with a wide variety of attitudes and behaviours (see for example their effects on charity behaviour studied by Sneddon et al. (2020); or climate action explored by Bouman et al. (2020)). To sum up, values motivate people to behave accordingly (Bardi and Schwartz, 2003). In the present study, we choose to explore the role of self-direction and universalism values in shaping students’ digital citizenship. Self-direction and universalism represent adjacent values for which Schwartz (2012) defines *joint motivational emphases*. Universalism is concerned with ensuring the welfare of others, whereas self-direction coupled with universalism, on the other hand, entails the belief that one should rely on one’s judgment and be comfortable with diversity and self-improvement (Beramendi and Zubieta, 2017). Generally, knowledge is considered an unproblematic phenomenon. As a result, the textbooks do not contain controversial topics or social conflicts; nor do they present clashing interpretations or viewpoints. However, described as a mix of skills to critical thinking, evaluate information, and making decisions (Puolimatka, 1995) is increasingly presented as one of the key outcomes of higher education programmes (Cruz et al., 2020; Bellaera et al., 2021). Therefore, university graduates as citizens must possess these skills to exert influence within their communities. Critical thinking becomes essential to develop digital citizenship where people become members of online communities and build collaborative and cooperative practices (Choi et al., 2017). Citizenship today requires individuals to express their views and critical thinking takes precedence over subservient accommodation. This refers to decision-making, shaping arguments, accepting other people’s views and choices, discussing them, shaping a personal perspective and making it public (Ten Dam and Volman, 2004), however, it also refers to “building relationships, autonomy and acceptance, access to services and supports, shared values and social roles and civic rights and responsibilities” (MacIntyre et al., 2021, p. 699) when defined by citizens themselves. Critical thinking represents a complex and debatable construct that differs from being a politically oriented educational objective (e.g., Giroux, 1992; McLaren, 1995; Moore, 2013; Larsson, 2017), leading to a higher level of thinking (e.g., Halpern, 1998; Schulz and FitzPatrick, 2016; Liu et al., 2021). In this theory, critical thinking is a key aspect of citizenship that allows citizens to engage in a pluralistic and democratic society and empowers them to influence that society. The common goal of critical thinking development and citizenship education is to encourage active participation in the community; respect and acknowledge one’s own self and others; develop social and moral values; establish values that consider divergent social viewpoints; practice listening and conflict resolution; and help maintain a safe environment. Critical thinking involves reasonable reflective thinking that aims to determine what to believe or how to act (Norris and Ennis, 1989; Ennis, 2018). A critical thinking process consists of three steps: recognising assumptions, articulating assumptions and evaluating their validity. Individuals need these skills to function effectively in a complex, democratic and modern society. In addition, higher education can help students develop their values and thinking skills through value clarification and fostering higher-order thinking abilities in personalised learning environments based on interactions with peers (Leming, 1998; Bezanilla et al., 2019; Lu et al., 2021).

## The present research

The growing interest in developing digital citizenship through education relates to the persistence of digital technologies in the social realm and the importance of information and digital literacy for personal development and social regulation (Milenkova and Lendzhova, 2021). Recently, several European institutions have designed effective training strategies for digital literacy and information (European Training Foundation, Turin, 2018; European Literacy Policy Network, 2020; European Commission, 2021). Digital citizenship becomes relevant in the context of online information processing, knowledge, online content creation and following a code of conduct for online behaviour. Many education aspects are relevant in supporting digital citizenship for students such as student learning and academic performance, student school environment and behaviour (Ribble and Bailey, 2007). Factors such as personal values and critical thinking skills may play an important part in the way people engage with novel information or behave and act in the virtual environment. For this reason, previous researchers have stressed personal values, such as collectivism, self-transcendence or self-enhancement (Sosik, 2005; Ahmad et al., 2021) and the role of critical thinking skills (Torney-Purta et al., 1999; Nguyen, 2012) in promoting digital citizenship behaviour. Previous research also underlines the predictive value of interpersonal communication competence for digital citizenship (see Xu et al., 2018) and the role of technology education in contrast with the non-significant effect of individual use of the Internet (Al-Abdullatif and Gameil, 2020).

Critical thinking is indispensable for a citizen to be truly able to exert influence in a community (Puolimatka, 1995, pp. 110–111); moreover, it is linked with communication skills and the capacity to influence others. Through digital citizenship, students have an opportunity to practice active and analytical information acquisition and to have an influence through different media. In the digital realm, students are no longer seen as passive receivers but as communicators with an active role. Living in an information society necessarily requires preparedness for critical thinking. In school, a student should be able to form questions and evaluate contradictory information as part of practicing the skills of a critical thinking citizen (Torney-Purta et al., 1999). Critical thinking skills are needed for students to reflect effectively on information and actions regarding citizenship (Halstead and Pike, 2006). Defined by Ennis (1985, p. 45) as a type of “reasonable, reflective thinking that is focused on deciding what to believe or do,” critical thinking refers to the way an individual interacts with novel information in terms of interpretation, analysis and evaluation. These abilities are valuable to recognise false assumptions and conclusions, see through bias and propaganda, use evidence impartially, assess the strengths and weaknesses of an argument and to draw justifiable conclusions that will shape the foundation of future actions. Such skills lie at the heart of responsible citizenship (Claire, 2001, pp. 112–114).

As argued above, some personal values have been explored in previous studies on digital citizenship, but to our knowledge self-direction and universalism yet to be included among them. Furthermore, although most conceptual frameworks for digital citizenship acknowledge the relevance of critical thinking, there is little empirical evidence to bridge these constructs (e.g., Herwati et al., 2020; Yildiz et al., 2020). The present research addresses these gaps and aims to show how students’ personal values and dispositions can help them self-regulate their learning process and support the development of the learning process in terms of digital citizenship. The digital citizenship concept reflects individual skills and competences to actively participate in the social arena. By reflecting a certain type of awareness of emergent social issues, digital citizenship is supported by the development of critical thinking and values as it involves taking responsibility for their position from different social perspectives. In this sense, digital citizenship promotes the positive development of individuals and communities (De Coster and Sigalas, 2017). The first goal of the present study is to explore the role of self-direction and universalism, values related to the enhancement of others, self-transcendence and reliance upon one’s own judgment (Beramendi and Zubieta, 2017) in shaping critical perspective towards online participation and the Internet as a measure of digital citizenship. The second goal is to determine the role of two critical thinking dispositions—learning orientation and cognitive integrity—in digital citizenship development (i.e., critical perspective towards online participation and the Internet). We suggest that learning orientation is relevant because it implies a disposition towards information-seeking as a personal strategy when solving a problem (Giancarlo et al., 2004). In addition, we propose that cognitive integrity is important because it implies a disposition towards interacting with contrasting perspective for the purpose of reaching the best decision (Giancarlo et al., 2004). The present study contributes to identifying the conditions under which the value–behaviour relationship is facilitated and informs the educational practice about personal resources that need to be advanced through learning for digital citizenship development. Considering the existing theoretical framework, we expected universalism and self-direction to predict critical perspective towards online participation and the Internet (respectively, Hypothesis 1 and Hypothesis 2). Consequently, learning orientation and cognitive integrity are expected to have a positive influence on critical perspective towards online participation and the Internet (respectively, Hypothesis 3 and Hypothesis 4). Further, universalism is expected to predict learning orientation (Hypothesis 5), while self-direction is expected to predict cognitive integrity (Hypothesis 6).

**Figure 1:**
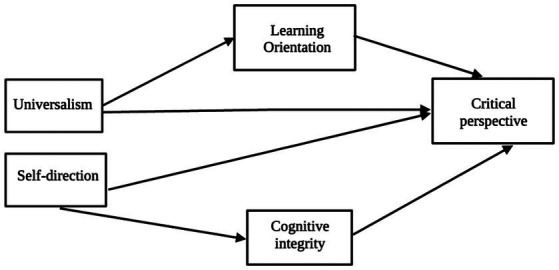
The hypothesised theoretical model.

The hypothesised model is depicted in Figure 1.

## Materials and methods

### Participants

The invitation to participate in this research reached 900 bachelor’s students enrolled in various social sciences domains such as psychology, human resources, special needs education, pedagogy and elementary education students. The computed response rate was 59.56%. The research sample size (*N* = 536) is considered appropriate for multivariate analysis (Hair et al., 2019). This cross-sectional study uses convenience sampling for its benefits in terms of costs and time, but it cannot be considered a representative sample. There were no exclusion criteria for the participants based on demographic variables. The student participants were selected from universities located in the north-eastern region of Romania. Participants completed an online survey after reading the informed consent statement on the first page. Participants received information regarding data security, the type of information being collected, data keeping and how their anonymity will be maintained. Participants were also informed that by completing the survey they were consenting to participate in the study. Further, they were instructed to save a copy of the document. Study participation was voluntary and anonymous. The research sample included 536 bachelor students aged 18 to 26 (*M* = 20.85, *SD* = 1.60). The large majority were women (92.16%) and 7.83% men. Out of the entire sample, 50.18% (*N* = 269) resided in urban areas while 49.81% (*N* = 267) resided in rural areas. One-third of the students were studying psychology. The participants’ characteristics are reported in Table 1.

**Table 1:** Participants characteristics.

Sample characteristics	*n*	%	*M*	*SD*
Age			20.85	1.60
*Gender*				
Female	494	92.16%		
Male	42	7.83%		
*Residing area*				
Rural	269	50.18%		
Urban	267	49.81%		
*Bachelor enrollment year*				
1st year	190	35.44%		
2nd year	115	21.45%		
3rd year	231	43.09%		
*Field of study*				
Psychology	175	32.64%		
Pedagogy	63	11.75%		
Elementary education	125	23.32%		
Special needs education	70	13.05%		
Human resources	103	19.21%		

### Procedure

The study received approval from the Research Ethics Committee of the university. The research took place during April and May 2022. The students received the information regarding the study in the classrooms. Later, the survey link was distributed to various university social media groups. The announcements related to this research included a link to the online survey form. In the first sections, participants were asked to read the informed consent form and provide demographic information. Before starting the study, the respondents were informed that participation was voluntary and they could withdraw from the study at any point. They also received information regarding data gathering, security and maintenance. The online survey was designed with a closed-answers interface for all variables, including demographic information. Because all responses were closed answers, there were no errors or missing data. All the answers had to be selected from a list and the form could not be submitted in the presence of a missing value. The questionnaire took around 30 min to complete. This study was carried out following the recommendations of the Code of Ethics of the university. The protocol was approved by the Ethics Committee for Research of the Faculty of Psychology and Educational Sciences. Following the Declaration of Helsinki, all participants gave written, informed consent for their participation in the study.

### Measures

The questionnaires were translated from English into Romanian using the forward–backward translation procedure (Hambleton et al., 1999). Afterwards, the translations were adjusted based on the back-translation process. The measures’ construct validity was explored through confirmatory factor analysis and the internal consistency was examined by McDonald’s omega (ω) reliability index.

#### Universalism and self-direction personal values

To assess universalism and self-direction personal values, specific scales of the Portrait Values Questionnaire (PVQ) were used (Schwartz et al., 2001). The instrument is based on Schwartz’s (1992) theory of human values and represents a novel and more concrete measurement method. The universalism scale includes six items and the self-direction scale contains four items. The respondents must rate their answers on a Likert-type scale from 1 (*not at all like me*) to 6 (*very much like me*). The universalism scale includes statements such as ‘*He/She thinks it is important that every person in the world be treated equally’*; ‘*He/She believes everyone should have equal opportunities in life’.* The self-direction scale includes items such as ‘*Thinking up new ideas and being creative is important to her’*; ‘*He/She likes to do things in her own original way’*.

#### Critical thinking dispositions: Learning orientation and cognitive integrity

To measure critical thinking dispositions, we used the learning orientation and cognitive integrity sub-scales from the California Measure of Mental Motivation (CM3) (Giancarlo et al., 2004). The learning orientation section comprises six items and the cognitive integrity section includes five reversed items rated on a four-point Likert-type scale from 1 (*strongly disagree*) to 4 (*strongly agree*). One item example for learning orientation is, ‘*I always look forward to learning challenging things’*; and for cognitive integrity, *‘It is just not that important to keep trying to solve difficult problems’.*

Digital citizenship was measured using the critical perspective towards online participation and the Internet sub-scale, part of the Digital Scale (Choi et al., 2017). It consists of seven items rated on a seven-point Likert-type scale ranging from 1 (*strongly disagree*) to 7 (*strongly agree*). Example items in this dimension include: ‘*I think online participation is an effective way to make a change to something I believe to be unfair or unjust’* or ‘*I think online participation promotes offline engagement’*.

### Data analysis

The data analysis procedure was supported by SPSS 26 software used for data recording and descriptive statistics analyses (i.e., means, standard deviations, skewness and kurtosis). Preliminary analyses were conducted to assess data normality. Investigation of the normal distribution of data is examined in terms of skewness (SK ≤ 3) and kurtosis (Ku ≤10) (Kline, 2011). Moreover, the Kaiser–Meyer–Olkin Measure of Sampling Adequacy (KMO ≥ 0.50) was used to investigate data and sample size adequacy for performing factor analysis (Kaiser, 1974). Bartlett’s test of sphericity was used to examine whether the correlation matrix is an identity matrix. A significant result (*p* < 0.050) indicates that the data is suitable for factor analysis (Snedecor and Cochran, 1989). The internal consistency or reliability between items was evaluated by McDonald’s omega reliability coefficient, which should have values above 0.65 (Creswell and Creswell, 2017).

In the first step, confirmatory factor analysis (CFA) was conducted to evaluate the construct validity of the measurement model (Brown, 2015). In the second step, the hypothesised structural equation model (SEM) was verified. SEM is a tool used in multicausal analysis at a given time in a theoretical structure, including observed and latent variables (Chonsalasin and Khampirat, 2022). The tested model included two latent exogenous variables (personal values: universalism and self-direction) and three endogenous variables (critical thinking dispositions: learning orientation and cognitive integrity and critical perspective towards online participation and the Internet). CFA and SEM were performed using IBM SPSS Amos 22 software. The goodness of fit of the model was assessed using the following indices: root means square error of approximation (RMSEA <0.08), values between 0.08 and 1 are considered marginal (Fabrigar et al., 1999), standardised root means square residual (SRMR <0.08), comparative fit index (CFI ≥ 0.90) and Tucker–Lewis’s index (TLI ≥ 0.90) (Bentler, 1990). The exogenous variables were allowed to correlate.

## Results

### Descriptive statistics and preliminary analyses

Descriptive statistics of the means, standard deviations, skewness, and kurtosis are presented in Table 2. The absolute values of skewness range from 1.900 to 0.079 (SK < 3) and the absolute values of kurtosis range from 0.03 to 3.29 (KU < 10), indicating that the data are normally distributed (Table 2). Further, the resulted values of KMO (KMO = 0.875) and Bartlett’s test of sphericity (*χ*^2^ = 4854.56, *df* = 378, *p* < 0.001) support the use of factor analysis.

**Table 2:** Descriptive statistics and results of CFA measurement model (*N =* 536).

Constructs	Items	*M*	*SD*	*SK*	*K*	Standardized loading	C.R. (*t*-value)	*R* ^2^
Universalism	val1	5.52	0.843	−1.900	3.290	0.472	9.773^***^	0.223
	val2	5.05	1.025	−0.865	0.143	0.514	10.590^***^	0.264
	val3	5.23	0.945	−1.028	0.185	0.729	14.511^***^	0.531
	val4	5.27	0.985	−1.262	1.007	0.709	–	0.503
	val5	5.24	0.963	−1.144	0.520	0.639	12.967^***^	0.408
	val6	4.90	1.165	−0.847	−0.036	0.575	11.771^***^	0331
Self-direction	val7	4.73	1.113	−0.537	−0.484	0.556	10.406^***^	0.320
	val8	5.20	0.960	−1.117	0.718	0.548	10.147^***^	0.300
	val9	5.13	0.978	−0.979	0.504	0.709	12.173^***^	0.503
	val10	5.24	0.985	−1.220	0.747	0.612	–	0.374
Learning Orientation	L1	3.71	0.503	−1.422	1.041	0.737	16.199	0.544
	L2	3.44	0.653	−0.761	−0.486	0.791	17.335	0.626
	L3	3.34	0.715	−0.656	−0.574	0.744	–	0.553
	L4	2.98	0.880	−0.382	−0.777	0.530	11.579	0.281
	L5	3.47	0.669	−0.968	0.075	0.709	15.584	0.503
	L6	3.36	0.742	−0.901	0.129	0.579	12.671	0.336
Cognitive integrity	cog1	2.68	0.920	−0.117	−0.852	0.237	4.716^***^	0.056
	cog2	2.64	0.966	−0.088	−0.982	0.442	8.520^***^	0.196
	cog3	3.35	0.807	−1.029	0.196	0.719	12.039^***^	0.517
	cog4	3.00	0.863	−0.474	−0.551	0.723	–	0.523
	cog5	3.02	1.016	−0.595	−0.900	0.568	10.553^***^	0.322
Critical perspective towards online use and the Internet	cp1	4.94	1.687	−0.483	−0.473	0.691	14.589^***^	0.477
	cp2	4.77	1.581	−0.368	−0.378	0.569	12.085^***^	0.324
	cp3	4.03	1.857	−0.079	−0.880	0.773	–	0.597
	cp4	4.50	1.769	−0.330	−0.679	0.567	11.921^***^	0.321
	cp5	4.47	1.658	−0.249	−0.515	0.613	12.940^***^	0.376
	cp6	3.54	2.005	0.186	−1.157	0.581	12.325^***^	0.337
	cp7	2.97	2.025	0.619	−0.978	0.529	11.213^***^	0.279

*M* = Mean, *SD* = standard deviation and ^***^ significant at *p* < 0.001.

McDonald’s ω of each subscale and construct is presented in Table 3. For universalism, McDonald’s ω = 0.769, for self-direction McDonald’s ω = 0.699, for learning orientation McDonald’s ω = 0.824, for cognitive integrity scale McDonald’s ω = 0.660 and for critical perspective and towards online participation and the Internet McDonald’s ω = 0.811 (Table 3). The values exceeded 0.65 threshold recommended by Creswell and Creswell (2017) and indicates that the measures have satisfactory internal consistency. This was confirmed by composite reliability (CR), where all construct values are between 0.777 and 0.881 (Table 3), whereas the general standard of CR should exceed 0.60 (Hair et al., 2019). Further, the average variance extracted (AVE) should be higher than 0.50 (Hair et al., 2019); here, the AVE values varied between 0.381 and 0.555 (Table 3). However, if AVE values are below the 0.50 threshold but the CR is greater than 0.60, then the construct’s convergent validity is satisfactory (Fornell and Larcker, 1981; Khampirat, 2021). Table 3 also shows the goodness of fit indices for all measurement models. The results of confirmatory factor analysis (CFA) showing an acceptable fit.

**Table 3:** Psychometric properties of the measures.

Constructs	Composite reliability (CR)	Average variance extracted (AVE)	McDonald’s omega	*χ2*	*df*	CFI	TLI	RMSEA	SRMR
Universalism	0.817	0.433	0.769	56.642	9	0.944	0.906	0.094	0.046
Self-direction	0.779	0.541	0.699	13.958	3	0.970	0.941	0.084	0.032
Learning orientation	0.881	0.555	0.824	44.209	9	0.969	0.948	0.086	0.036
Cognitive integrity	0.777	0.425	0.660	12.345	5	0.982	0.964	0.052	0.028
Critical perspective	0.796	0.381	0.811	68.757	13	0.950	0.920	0.090	0.044

### Measurement model results

The CFA examined the 5 latent and 28 observed variables. All latent variables were allowed to correlate with each other (Anderson and Gerbing, 1988). The measurement model was examined using the maximum-likelihood method, which indicated a satisfactory fit to the data as follows: *χ*^2^ = 766.518, *df* = 339, *p* < 0.001, CFI = 0.906, TLI = 0.899, RMSEA = 0.040 (90% [CI]: 0.04 to 0.05), SRMR = 0.050. Likewise, as Table 2 shows, the values of standardised loading of the 28 indicators vary between 0.237 and 0.791 and have statistical significance (*p* < 0.001), which confirms convergent validity. Further, the measurement model was used to test the hypothetical structural model.

### Structural equation model results

**Figure 2:**
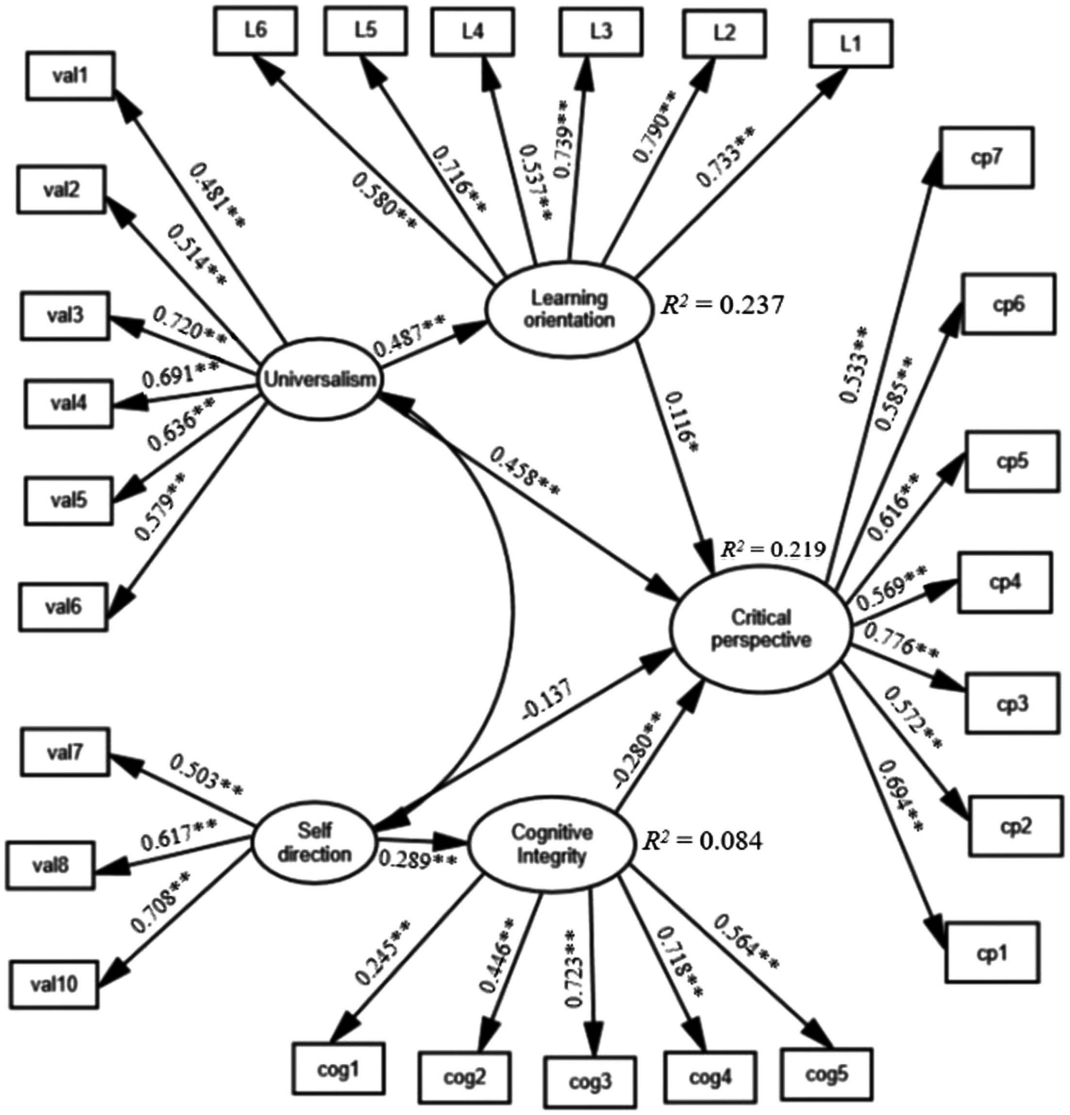
The structural equation model. All coefficients are standardized. *p <0.05, **p <0.001.

The SEM results of higher education students’ digital citizenship suggested that the third item of the self-direction scale should be deleted due to second-order factor cross-loading. Also, two items of the digital citizenship subscale were allowed to correlate among them. Consequently, the goodness of fit indices for the SEM model are as follows: *χ*^2^ = 744.792, *df* = 316, *p* < 0.001, RMSEA = 0.050, CFI = 0.900, TLI = 0.889 and SRMR = 0.061. These values indicate that the model has an acceptable fit (see Figure 2). The significance level of the hypotheses was examined by computing standard beta (β) values for each relationship (Figure 2; Table 4).

**Table 4:** The results of the structural model.

Hypothesis path	Standardized estimate	C.R. (*t*-value)	Value of *p*
H1: Universalism → Digital citizenship	0.458	3.648	0.000
H2: Self-direction → Digital citizenship	−0.137	−1.096	0.273
H3: Learning orientation → Digital citizenship	0.116	1.991	0.046
H4: Cognitive integrity → Digital citizenship	−0.280	−4.821	0.000
H5: Universalism → Learning orientation	0.487	8.663	0.000
H6: Self-direction → Cognitive integrity	0.289	4.784	0.000

→ regression on.

High and significant beta (*β*) values highlight the substantial effects of endogenous latent variables. Further, to investigate the significance of the beta values, the critical ratios (t-values) method was used. The results show that universalism has a positive significant effect on critical perspective towards the Internet and online participation (Hypothesis 1: *β* = 0.458, C.R. = 3.648, *p* = 0.000), showing a positive association between the two variables. In contrast, self-direction showed no significant effect the digital citizenship measure (Hypothesis 2: *β* = −0.769, C.R. = −1.096, *p* = 0.273). Therefore, the second hypothesis is not supported. The third hypothesis documented a significant positive relationship between learning orientation and critical perspective towards the Internet and online participation, and the results supported this hypothesis (Hypothesis 3: *β* = 0.116, C.R. = 1.991, *p* = 0.046). A positive effect of cognitive integrity on the digital citizenship measure was expected. In turn, the results show a negative effect of cognitive integrity (Hypothesis 4: *β* = −0.280, C.R. = −4.821, *p* < 0.001). Finally, personal values, universalism and self-direction predicted the hypothesised direction for the critical thinking dispositions learning orientation (Hypothesis 5: *β* = 0.487, C.R. = 8.663, *p* < 0.001) and cognitive integrity (*β* = 0.289, C.R. = 4.784, *p* < 0.001).

## Discussion and conclusion

The present study aimed to explore the role of personal resources in supporting learning effectiveness related to the development of digital citizenship as a transversal competence in higher education by examining the role of two adjacent values, self-direction and universalism. Furthermore, we examined the mediating role of students’ critical thinking disposition, such as learning orientation and cognitive integrity. Modern society has encouraged citizenship to also expand into the digital environment. Yet, for digital citizenship to develop, personal values must be clearly defined and critical thinking dispositions must be put into action concerning digital knowledge. Sustainable digital citizenship can be created by including personal values in virtual communities (Ghosn-Chelala, 2019). Values are empirically associated with a wide variety of attitudes (Bardi and Schwartz, 2003) and behaviours that relate also to critical thinking dispositions and are highly valued in the digital space. The present findings could contribute to a better understanding of the personal resources that can support the digital citizenship development process. The first hypothesis presumed that values linked to individual action and the enhancement of others, namely self-direction and universalism, would be positively related to the critical perspective towards online participation and the Internet as a measure of digital citizenship. The results show that higher universalism relates to sustainable digital citizenship. These findings are in line with previous research that universalism relates to actions and behaviours that promote the welfare of others (Bardi and Schwartz, 2003; Stankevičiūtė and Wereda, 2020). This shows that the universalism value is an important resource for developing citizenship in the spirit of fostering a critical perspective towards online participation and the Internet. The second hypothesis which focused on the positive effect of self-direction was not supported by the results. The third and fourth hypotheses focused on the role of critical dispositions, learning orientation and cognitive integrity in supporting digital citizenship development. Higher learning orientation motivates individuals towards intellectual activities that involve reasoning, particularly wanting to expand one’s knowledge and using information-seeking strategies when attempting to solve a problem (Giancarlo et al., 2004). Therefore, individual orientation towards information-seeking endorses digital citizenship and a critical perspective towards online participation and the Internet. Surprisingly, cognitive integrity had a negative effect on digital citizenship as a critical perspective towards online participation and the Internet. Because critical integrity is defined within this study as the “disposition toward interacting with differing viewpoints for the sake of learning the truth or reaching the best decision” and “valuing the fair-minded consideration of alternative perspectives” (Giancarlo et al., 2004, p. 353), its high level may foster the consideration of offline or traditional means of participation and thereby lower engagement with digital citizenship. In other words, in relation with digital citizenship it may function as a blocker at high levels and as an enhancer at low levels; however this needs to be further explored in future studies. The last two hypotheses show the effect of personal values on critical thinking dispositions. Hence, individuals with higher universalism reported higher learning orientation. Focusing on the welfare of others motivates individuals towards intellectual activities that involve reasoning, particularly wanting to expand one’s knowledge and using information-seeking strategi es when attempting to solve a problem (Giancarlo et al., 2004). Similar results were reported by Coskun and Altinkurt (2016), showing that values like universalism support critical thinking dispositions in students. However, for critical thinking not to descend on the reasoning that holds logically valid arguments founded on unreasonable or unethical premises, an explicit underpinning in values is needed (Higgins, 2014). These findings have relevance for both theory and practice. From a theoretical perspective, this is one of the few studies examining the implications of values and critical thinking dispositions in the context of digital citizenship. Thus, it expands the conceptual model of value-related behaviour across the virtual domain. From an educational practice perspective, the results show that by supporting the development of certain personal values and critical thinking dispositions may support the development of digital citizenship. Higher education educational practices should stimulate the development of specific values in students. At the same time, students should acquire skills and dispositions that enable them to think critically and to analyse various opinions on their value orientation. Therefore, teaching strategies should combine strategies for advancing the development of specific values by teaching students to think critically. Teachers stimulate these values *via* subject matter, chosen examples and reactions to their students (Veugelers, 2010). Teachers can express values implicitly in the hidden curriculum (Giroux and Purpel, 1983) or by means of reflection; they can also be explicit about the values they express and the way they express them (Liston and Zeichner, 1991). In interpreting these findings, some limitations should be noted. First, our research is limited to only one factor of digital citizenship, the critical perspective towards online participation and the Internet, and does not investigate the larger spectrum of behaviours in the digital space. Second, using a cross-sectional design prevents us from drawing any inferences regarding the causality of the relationships between self-direction and universalism values, critical thinking dispositions and digital citizenship. In addition, generalisability is limited by the sample characteristics of mostly young and well-educated females. Although the sample size was adequate due to convenience sampling, the results cannot be generalised beyond young adult females. Hence, future research should endeavour sampling a balanced ratio of men and women. Furthermore, to minimise measurement errors, more extensive measures of digital citizenship should be applied. Despite these limitations, this study expands the role of values and highlights its importance related to critical thinking dispositions. Using a cross-sectional design, the results show that universalism value, learning orientation and cognitive integrity predict critical perspective towards online participation and the Internet. We believe that these findings have important educational implications and may substantiate a mechanism that can advance digital citizenship in youth.

## Data availability statement

The raw data supporting the conclusions of this article will be made available by the authors, without undue reservation.

## Ethics statement

The studies involving human participants were reviewed and approved by Ethics Committee for Research of the Faculty of Psychology and Educational Sciences. The patients/participants provided their written informed consent to participate in this study.

## Author contributions

GA, NP, and MM: conceptualisation, methodology, validation, formal analysis, data curation, and writing–original draft preparation. GA: visualisation and funding acquisition. All authors contributed to the article and approved the submitted version.

## Funding

This work was supported by a grant of the “Alexandru Ioan Cuza” University of Iasi, within the Research Grants program, Grant UAIC, code GI-UAIC-2021-03.

## Conflict of interest

The authors declare that the research was conducted in the absence of any commercial or financial relationships that could be construed as a potential conflict of interest.

## Publisher’s note

All claims expressed in this article are solely those of the authors and do not necessarily represent those of their affiliated organizations, or those of the publisher, the editors and the reviewers. Any product that may be evaluated in this article, or claim that may be made by its manufacturer, is not guaranteed or endorsed by the publisher.
